# Outcomes following dialysis and transplantation in amyloidosis: ANZDATA analysis

**DOI:** 10.1186/s12882-026-05230-3

**Published:** 2026-08-03

**Authors:** Sumedh Jayanti, Dharshana Sabanayagam, Joshua L. Lee, Katrina Chau, Bhadran Bose, Brian J. Nankivell, Fiona Kwok, Jennifer S. Y. Li

**Affiliations:** 1 Department of Renal Medicine and Transplant, Westmead Hospital, Sydney, NSW Australia; 2 The University of Sydney, Sydney, NSW Australia; 3 Children’s Hospital at Westmead Centre for Kidney Research, Westmead, NSW Australia; 4 Centre for Transplant and Renal Research, Westmead Institute for Medical Research, 176 Hawkesbury Road, Westmead, NSW 2145 Australia; 5 Department of Renal Medicine, Blacktown & Mount Druitt Hospital, Sydney, NSW Australia; 6 Department of Renal Medicine, Nepean Hospital, Sydney, NSW Australia; 7 The George Institute for Global Health, Sydney, NSW Australia; 8 Department of Haematology, Westmead Hospital, Sydney, NSW Australia; 9 Western Sydney University, Sydney, NSW Australia

**Keywords:** Amyloidosis, Kidney failure, Kidney replacement therapy, Dialysis, Transplant

## Abstract

**Background:**

The kidneys are frequently affected by systemic amyloidosis. Despite this, contemporary survival outcomes for patients reaching kidney failure due to amyloidosis remain poorly defined relative to other causes of kidney failure. This study aims to characterise patient survival with renal amyloidosis requiring kidney replacement therapy (KRT) and directly compare these outcomes with diabetic nephropathy, glomerular disease, and polycystic kidney disease (PKD).

**Methods:**

This was a retrospective cohort study using the Australia and New Zealand Dialysis and Transplant Registry for patients who initiated KRT between 1990 and 2022. A propensity-matched analysis was performed at 1:4 ratio for amyloidosis versus PKD, glomerular disease and diabetes. Patients were matched for age, sex, treatment era, comorbidities and dialysis vintage. Kaplan-Meier survival curves and Cox proportional hazards were used to compare patient survival on dialysis and post-transplant.

**Results:**

84,148 incident dialysis patients were included, of which 654 (0.8%) had kidney failure attributed to amyloidosis. Using amyloidosis as the reference group, matched comparator cohorts had lower mortality hazards: diabetic nephropathy (HR 0.68, 95% CI 0.62–0.74), glomerular disease (HR 0.38, 95% CI 0.34–0.42), and PKD (HR 0.28, 95% CI 0.26–0.32). 23,463 incident transplant recipients were included, of which 48 (0.2%) had kidney failure secondary to amyloidosis. Using amyloidosis as the reference group, post-transplant mortality hazards were similar for T2DM (HR 1.00, 95% CI 0.75–1.35), but lower for T1DM (HR 0.72, 95% CI 0.52–0.98), glomerular disease (HR 0.40, 95% CI 0.28–0.57), and PKD (HR 0.38, 95% CI 0.29–0.56). Amyloidosis subtype data were unavailable, limiting assessment of disease-specific outcomes.

**Conclusions:**

Kidney failure due to amyloidosis was associated with markedly worse survival on dialysis and less favourable post-transplant patient survival compared with several comparator kidney diseases. However, death-censored graft survival appeared broadly comparable, suggesting that excess post-transplant risk is largely driven by patient mortality rather than intrinsic graft failure.

**Supplementary Information:**

The online version contains supplementary material available at https://doi.org/10.1186/s12882-026-05230-3.

## Background

Amyloidosis comprises a heterogeneous group of disorders characterised by the extracellular deposition of misfolded protein, with kidney involvement being a major contributor to morbidity and mortality. There are many different types of amyloidosis and each with varying incidence of kidney involvement and rate of progression to kidney failure. Existing literature on outcomes for patients with amyloidosis receiving kidney replacement therapy (KRT) largely comprises small, single-centre series or registry-based studies. Registry studies conducted most prior to 2020 that treat amyloidosis as a single entity demonstrate higher risks of death and graft loss when compared with other kidney diseases, with a median post-transplant survival of approximately 5–6 years versus over a decade in non-amyloid kidney failure [1–3]. Subtype-specific studies highlight the heterogeneity of outcomes between subtypes but are limited by small sample size. For example, survival on dialysis is particularly poor for light chain (AL) amyloidosis patients, often less than 24 months [4, 5], although kidney transplantation offers improved outcomes in specialised centres, especially for patients achieving a complete haematologic response [6–9]. These studies also do not reflect outcomes in a contemporary cohort where the use of daratumumab-based regimens have improved outcomes for patients with AL amyloidosis [10]. In Amyloid A (AA) amyloidosis, dialysis survival is similarly poor [5], though selected cohorts demonstrate comparatively superior survival and favourable transplant outcomes when the underlying inflammation is well-controlled [11–14]. For hereditary forms such as Fibrinogen Aα-chain (AFib) amyloidosis, patients can have a long survival post-transplant but with high recurrence rates [13, 15–18]. These heterogeneous findings underscore the need for contemporary, adjusted comparisons with the broader dialysis and transplant population. These studies are summarised in Supplementary Tables 1 and Supplementary Fig. 1.

A major gap in existing literature is that few studies have adequately adjusted outcome estimates for patients with amyloidosis and directly compared these with more common causes of kidney failure. Without appropriate adjustment, existing estimates may therefore misrepresent the true attributable risk of amyloidosis on dialytic and transplant outcomes.

To address this gap, this study uses comprehensive national registry data from the Australia and New Zealand Dialysis and Transplant (ANZDATA) Registry and applies propensity score matching to directly compare dialysis and transplant outcomes of patients with kidney failure due to amyloidosis against those with other major kidney diseases. By controlling for demographic, clinical, and temporal variables, this study provides robust comparative assessment of long-term outcomes on dialysis and following transplantation in this rare but clinically significant population.

## Methods

### Study population

**Fig. 1:**
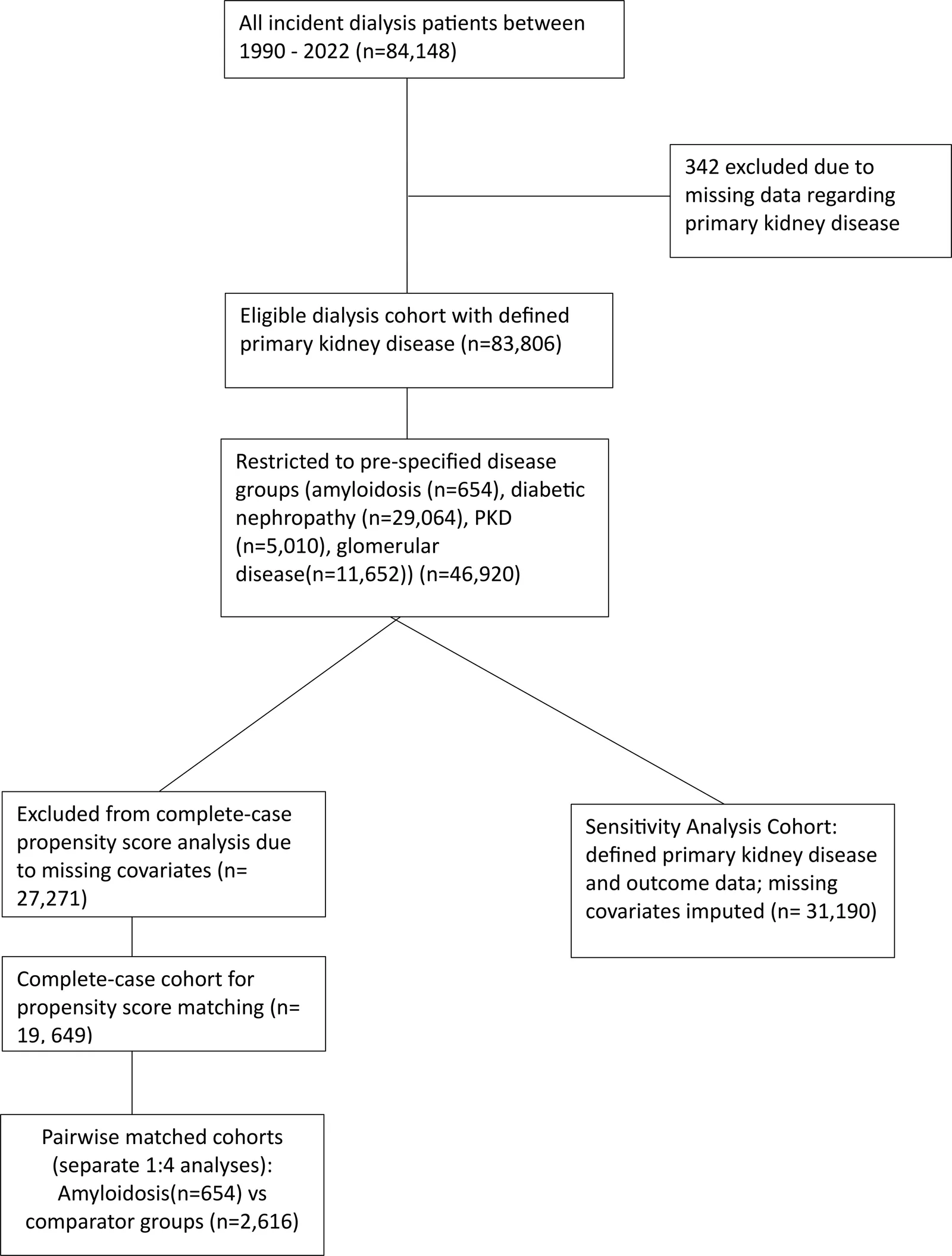
STROBE-consistent cohort flow diagram for the ANZDATA dialysis cohort. The figure shows derivation of the eligible dialysis cohort, restriction to prespecified comparator disease groups, exclusions for complete-case propensity score matching, final pairwise matched cohorts, and the cohort used for multiple-imputation sensitivity analyses. PKD, polycystic kidney disease; PSM, propensity score matching. Sensitivity cohort includes patients with defined primary kidney disease and outcome data, excluding those with missing/non-imputable covariates required for the adjusted model

Data from the ANZDATA registry were used to identify patients aged 18 years and over who commenced (1) dialysis as their first form of KRT and (2) those who had undergone their first kidney transplant between the 1st of January 1990 and the 31st of December 2022. Patients who started dialysis were followed from dialysis initiation to death, recovery of kidney function, until kidney transplantation, or 31st December 2022, whichever came first. Patients who received a kidney transplant were followed from the time of transplantation until death, graft loss, or 31st December 2022, whichever came first. Patients under the age of 18 years old and re-grafts were excluded. Patients with missing primary kidney disease were excluded from all analyses. The primary propensity score-matched analyses were performed using complete-case data for variables included in the propensity score model and for outcome ascertainment. Patients with missing matching covariates were excluded from the complete-case matched analyses but were retained in the multiple-imputation sensitivity analyses where eligible. The derivation of the complete-case and multiple-imputation analytic cohorts is shown in Figs. 1 and 3. The study was approved by both Western Sydney Local Health District Human Research Ethics committee (2024/ETH01170) and the ANZDATA executive.

### Exposure

The primary exposure was the cause of kidney failure. For the primary analysis the dialysis cohort was categorised into the following causes of kidney failure: amyloidosis, diabetic nephropathy, glomerular disease and polycystic kidney disease (PKD). For the transplant cohort, the cause of kidney failure was categorised as amyloidosis, diabetic nephropathy due to type 1 diabetes mellitus (T1DM), diabetic nephropathy due to type 2 diabetes mellitus (T2DM), glomerular disease and PKD. The subtype of amyloidosis is not recorded in the ANZDATA registry. Glomerular disease included those recorded as ‘glomerulonephritis – no histology’, ‘nephrotic syndrome’, ‘minimal change nephropathy’, ‘IgA nephropathy’, ‘lupus nephritis’, ‘minimal change disease’, ‘membranous nephropathy’, ‘FSGS’, ‘mesangiocapillary glomerulonephritis’, ‘systemic vasculitis’, ‘ANCA vasculitis’, ‘Anti-GBM disease’ and ‘cryoglobulinaemia’.

These groups were chosen for comparison as patients with diabetic nephropathy are known to have poor outcomes on dialysis and post-transplant whereas those with PKD and glomerular disease tend to perform better [19–21]. Transplant recipients with diabetic nephropathy were further stratified into T1DM and T2DM to assess for the possible survival benefit of simultaneous pancreas-kidney transplantation [22].

### Covariates

The ANZDATA registry provided demographic data, cause of kidney disease, key comorbidities (diabetes, hypertension, smoking, chronic lung disease, peripheral vascular disease and coronary artery disease), KRT details (date commenced, modality) and outcome data (death, graft loss, transplant, disease recurrence).

### Outcomes

The primary outcome for the dialysis cohort was all-cause mortality. The primary outcomes for the transplant cohort were all-cause mortality, all-cause graft survival, death-censored graft survival (defined as graft loss excluding death with a functioning graft) and recurrence of primary kidney disease.

### Statistical analysis

Demographic and comorbid characteristics were expressed as frequency (and percentages) for categorical variables, mean ± standard deviation for continuous variables. Comparisons between exposure groups were analysed using chi-square tests and unpaired t-tests.

Using complete-case data, propensity-score matching was performed for amyloidosis versus diabetic nephropathy, glomerular disease, or PKD at 1:4 ratio using R statistical software (version 4.2.2) and the “designmatch” package (Zubizaretta et al., version 0.5.4) [23]. Propensity score models for dialysis patients included sex, dialysis era (successive decades 01/01/1990 to 31/12/2000, 01/01/2001 to 31/12/2010, 01/01/2011 to 31/12/2022), smoking status, chronic lung disease, coronary artery disease, peripheral vascular disease, initial dialysis modality and age starting dialysis. Propensity score models for transplant patients included sex, transplant era (successive decades 01/01/1990 to 31/12/2000, 01/01/2001 to 31/12/2010, 01/01/2011 to 31/12/2022), smoking status, chronic lung disease, coronary artery disease, peripheral vascular disease, age at transplantation, donor type and dialysis duration until transplantation. Diabetes was not used as a variable because it would be skewed to the diabetic nephropathy group. Bipartite cardinality matching was performed to identify optimal 1:4 matches and fine balance was used to match categorical variables and mean constraint to balance continuous variables using absolute standardised differences (cutoff < 0.1). Because comparisons were conducted independently, the same amyloidosis patient could contribute to more than one matched analysis across comparator groups. Matching was performed without replacement within each pairwise comparison, meaning that each comparator patient could only be selected once as a matched control in that analysis.

The association between cause of kidney failure and primary outcomes were examined using Kaplan-Meier survival curves and a cause-specific Cox proportional hazards model, with competing events treated as censored. In the dialysis cohort, kidney transplant was considered a competing event. In the transplant cohort, for all-cause mortality, return to dialysis post-graft loss was considered a competing event, and for all-cause graft survival, death was considered a competing event. For death-censored graft survival, deaths were treated as censoring events. The estimates were reported as cause-specific hazard ratio (cs-HR) and 95% confidence intervals (95% CI). Amyloidosis was the reference group with a HR below 1 indicating a lower hazard in the comparator group relative to amyloidosis.

As a sensitivity analysis, we compared outcomes in amyloidosis patients with the full unmatched cohort of patients with kidney failure due to diabetic nephropathy, glomerular disease and PKD. Missing values were imputed using multiple imputation by chained equations (R mice package [24]) under the assumption of missing at random for covariates with less than 10% missing data (Table S2). Five imputed datasets were created, incorporating sex, donor type, smoking status, coronary artery disease, chronic lung disease, and peripheral vascular disease. Immunosuppression details were reported descriptively in the demographic data and not as a factor in the analyses given there was > 20% missing data for immunosuppression types and lack of therapeutic drug levels or doses. We then used an adjusted cause-specific Cox proportional hazard model to examine the association between cause of kidney failure and primary outcomes. Proportional hazards assumptions were assessed using Schoenfeld residuals and visual inspection of scaled residual plots. Competing events were treated as censored and the same covariates as in the propensity-score model were adjusted for. We also performed additional competing-risk analyses using a Fine and Gray model, because these models estimate associations with cumulative incidence in the presence of competing risks, using the proportional hazards model for the sub-distribution to estimate the sub-hazard ratios (SHRs). In the dialysis cohort, kidney transplant was considered a competing event. In the transplant cohort, for all-cause mortality, return to dialysis post-graft loss was considered a competing event, and for all-cause graft loss, death was considered a competing event. A two-sided p-value < 0.05 was considered statistically significant.

R statistical software (R Foundation for Statistical Computing, Version 4.2.2, Boston, USA) was used for all statistical analyses.

## Results

### Dialysis cohort from ANZDATA registry

#### Baseline characteristics

A total of 84,148 incident dialysis patients were identified within the study period, with a median follow up time of 5.9 years (IQR: 3.8–7.4 years). 342 dialysis patients (0.40%) were excluded due to missing data regarding their primary kidney disease (Fig. 1). Baseline characteristics of the unmatched and matched cohorts are shown in Table 1. In the unmatched cohort, a total of 654 (0.77%) patients had kidney failure attributed to amyloidosis, and compared to the non-amyloid cohort, they were older at time of dialysis commencement but had lower incidence of coronary artery disease, peripheral vascular disease and diabetes. In the matched cohort, there is an expected imbalance of comorbid diabetes in amyloidosis versus the diabetic cohort due to the nature of the comparison.

**Table 1:** Baseline characteristics of dialysis patients in the ANZDATA registry grouped by cause of kidney disease

Dialysis cohort characteristics	Propensity match for kidney failure vs. amyloidosis							
	Amyloidosis (*n* = 654)	Non-Amyloidosis (*n* = 83,494)	Diabetes (*n* = 2616)	SMD	PKD (*n* = 2616)	SMD	Glomerular Disease (*n* = 2616)	SMD
Sex								
Male	365 (56)	50,765 (61)	1562 (60)	0.016	1499(57)	0.044	1615 (62)	0.072
Female	289 (44)	32,729 (39)	1054 (40)		1117 (43)		1001 (38)	
Dialysis Era				0.002		0.016		0.037
1990–2000	149 (23)	17,095 (20)	579 (22)		642 (25)		504 (19)	
2001–2010	226 (35)	25,489 (31)	928 (36)		810 (31)		931 (36)	
2011–2022	279 (43)	40,910 (49)	1109 (42)		1164 (45)		1181 (45)	
Age at dialysis (years)	64.8 +/- 17.1	59.4 +/-15.2	65.0 +/- 10.9	0.03	63.8 +/- 12.5	0.026	65.2 +/- 11.1	0.032
Dialysis modality^	186 (28)	23,636 (28)	737 (28)	0.002	793 (30)	0.063	664 (25)	0.09
Smoking History*	286 (44)	45,059 (54)	1255 (49)	0.03	1101 (42)	0.05	1057 (40)	0.07
Chronic Lung Disease	125 (19)	13,250 (16)	381 (15)	0.09	332 (13)	0.046	504 (19)	0.056
Coronary Artery Disease	196 (30)	31,448 (38)	852 (33)	0.01	812 (31)	0.04	818 (31)	0.005
Peripheral Vascular Disease	88 (13)	19,563 (23)	908 (35)	0.04	324 (12)	0.009	397 (15)	0.07
Diabetes	75 (11)	38,752 (46)	2616 (100)	N/A	288 (11)	N/A	511 (20)	N/A

Expressed as n (%) for categorical variables or mean (standard deviation) for continuous variables. Standardised mean differences (SMD) compare amyloidosis with each propensity score-matched comparator cohort. Absolute SMD values < 0.10 were considered indicative of acceptable covariate balance. For categorical variables with more than two levels, the SMD shown represents the maximum absolute SMD across categories. N/A outlines covariates that propensity score matching was not conducted for

*Smoking includes current or former smokers. ^Peritoneal Dialysis as initial modality (vs. haemodialysis)

#### Survival analysis

**Fig. 2:**
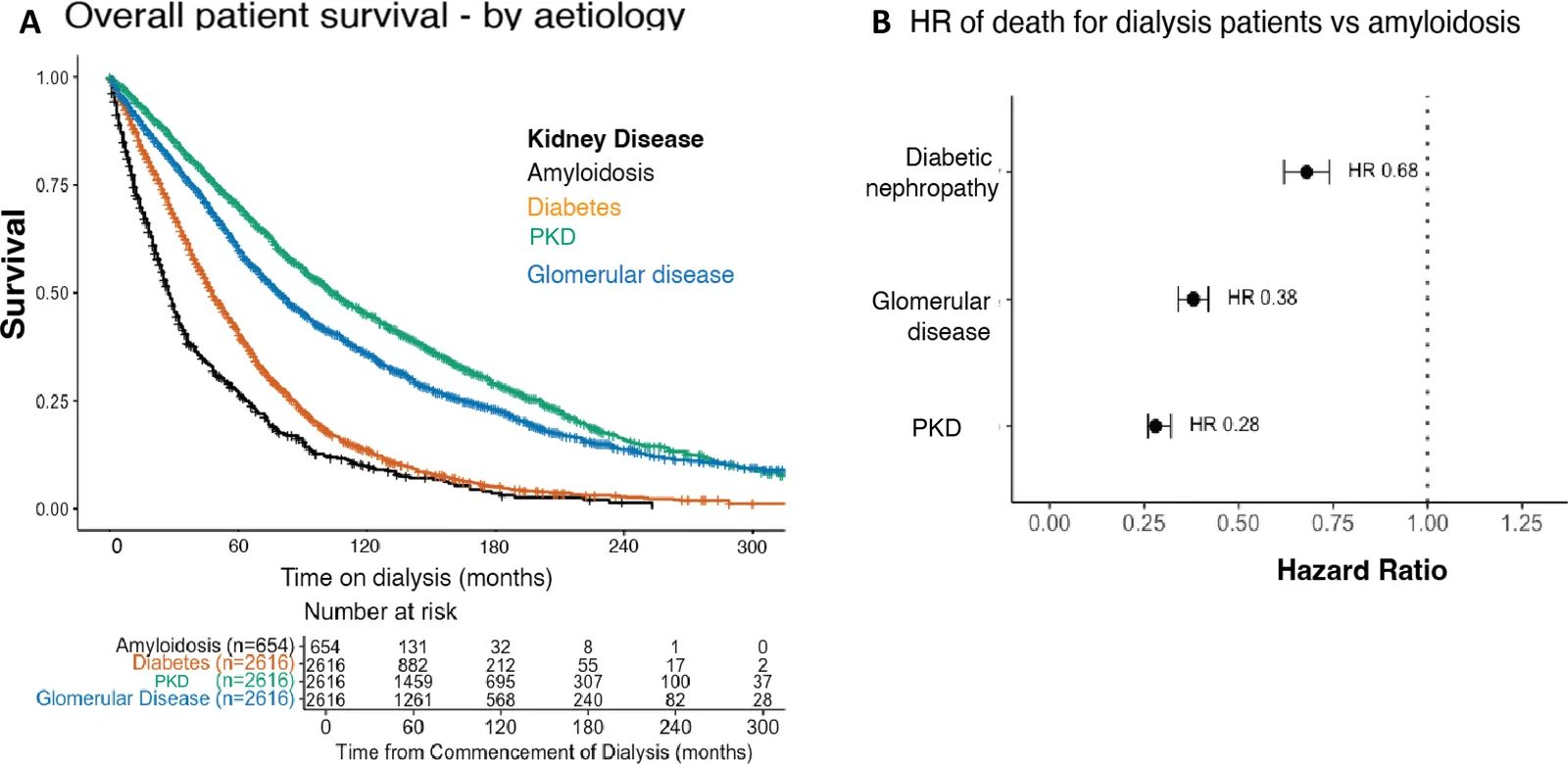
(**A**) Kaplan Meier survival curve comparing survival between propensity-score matched cohorts of amyloidosis, diabetic, glomerular disease and PKD dialysis patients (**B**) Hazard ratio for mortality for amyloidosis compared to diabetic nephropathy, glomerular disease and PKD. Amyloidosis is the reference group. HRs < 1 indicate lower hazard in the comparator group relative to amyloidosis

The median survival on dialysis was 27 months (95% CI: 25–30 months). Compared with matched cohorts, dialysis patients with amyloidosis had worse survival than those with diabetic nephropathy (HR, 95% CI) (0.68, 0.62–0.74), glomerular disease (0.38, 0.34–0.42) and PKD (0.28, 0.26–0.32) (Figs. 2A, B).

**Fig. 3:**
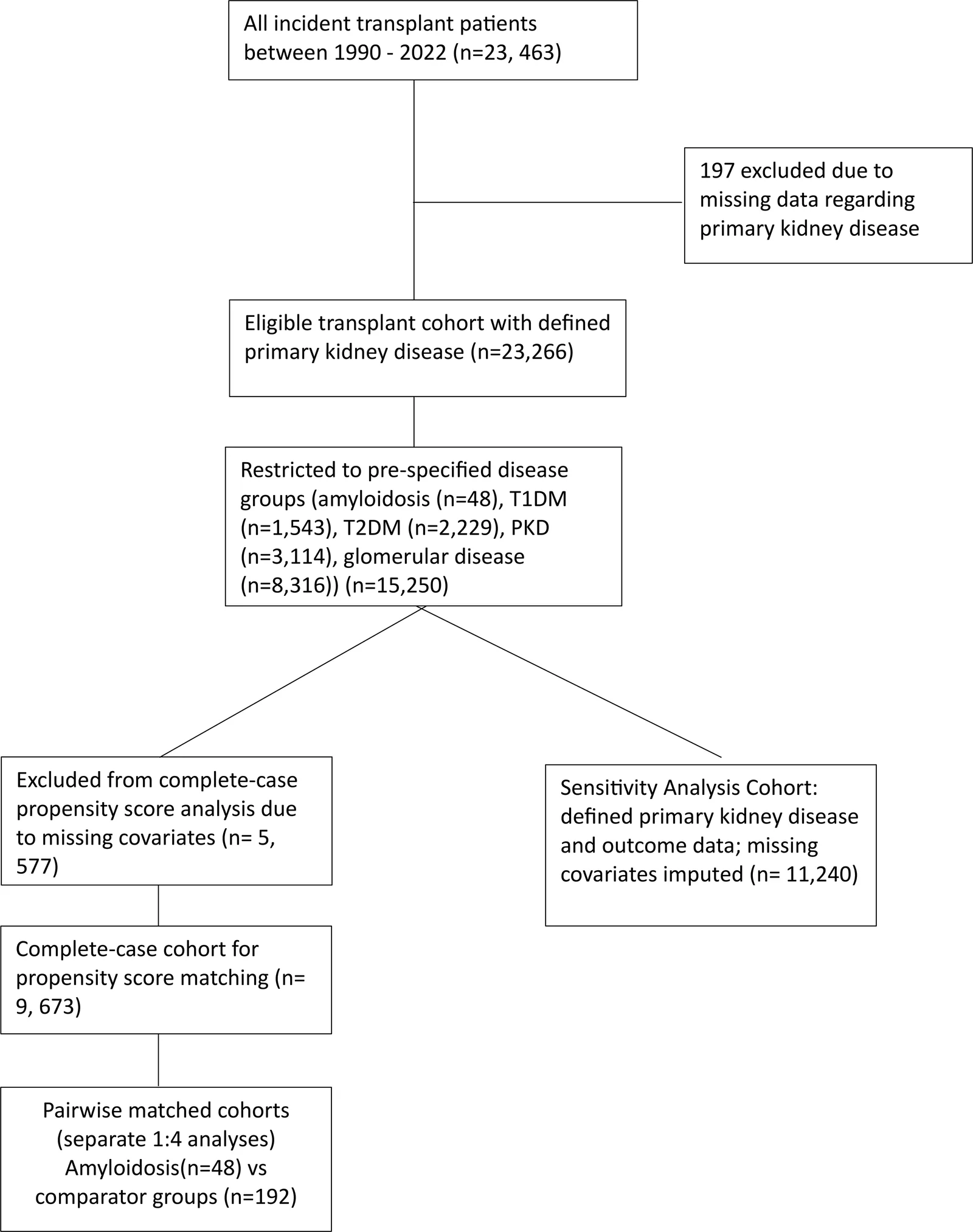
STROBE-consistent cohort flow diagram for the ANZDATA transplant cohort. The figure shows derivation of the eligible transplant cohort, restriction to prespecified comparator disease groups, exclusions for complete-case propensity score matching, final pairwise matched cohorts, and the cohort used for multiple-imputation sensitivity analyses. PKD, polycystic kidney disease; T1DM, type 1 diabetes mellitus; T2DM, type 2 diabetes mellitus; PSM, propensity score matching. Sensitivity cohort includes patients with defined primary kidney disease and outcome data, excluding those with missing/non-imputable covariates required for the adjusted model

The causes of death in dialysis patients with amyloidosis are reported in Table S3. The most common causes of death are cardiovascular disease and dialysis withdrawal, similar to the non-amyloid population.

Survival analyses comparing dialysis patients with kidney failure due to amyloidosis and the rest of the dialysis cohort are shown in Fig. S2 and Table S5.

#### Sensitivity analysis

Sensitivity analyses confirmed that overall survival on dialysis was lower in the amyloidosis cohort compared to the whole cohorts of patients with diabetic nephropathy (HR, 95% CI) (0.65, 0.57–0.74), glomerular disease (0.35, 0.30–0.42) and PKD (0.29, 0.26–0.32), with similar results in the Fine and Gray model (Table 2). Some evidence of non-proportionality was observed, likely reflecting changes in relative mortality hazards over the long study period and the high early mortality risk in amyloidosis. No major violation was identified that changed the direction of the primary disease-group associations; Cox hazard ratios were therefore interpreted as average associations over follow-up.

**Table 2:** Sensitivity analysis: cox and competing-risk models comparing mortality in dialysis patients by cause of kidney failure, using amyloidosis as the reference group

Model	Diabetic nephropathy		Glomerular disease		PKD	
		*p*-value		*p*-value		*p*-value
Multivariable (cs-HR)	0.65 (0.57–0.74)	< 0.001	0.35 (0.30–0.42)	< 0.001	0.29 (0.26–0.32)	< 0.001
Competing Risks* (sHR)	0.71 (0.63–0.77)	< 0.001	0.42 (0.35 to 0.49)	< 0.001	0.30 (0.21 to 0.37)	< 0.001

Data is expressed as cause-specific hazard ratio (cs-HR) or subdistribution hazard ratio (sHR) and 95% confidence interval (CI). Adjusted for sex, dialysis era, age at dialysis commencement, dialysis modality, smoking history, chronic lung disease and peripheral vascular disease. *Fine and Gray model, competing events of transplant. Amyloidosis is the reference group. HRs < 1 indicate lower hazard in the comparator group relative to amyloidosis

### Kidney transplant cohort from the ANZDATA registry

#### Baseline characteristics

From the ANZDATA registry, 23,463 incident kidney transplant recipients were identified during the study period with a median follow up time of 11.2 years (IQR: 8.4–13.2 years). Forty-eight (0.2%) patients had kidney failure due to amyloidosis. 197 transplant recipients (0.8%) were excluded due to missing data regarding their primary kidney disease (Fig. 3). Baseline characteristics of the unmatched and matched cohorts are shown in Table 3. Patients with amyloidosis were more likely to be older at transplantation, transplanted after 2010 (30 out of 48), have less comorbid diabetes and fewer were on cyclosporine for immunosuppression at time of transplantation. 24% of the matched T1DM cohort had a multi-organ transplant, which is most likely to be an SPK transplant. The median wait-time on dialysis to receiving a transplant was 28.4 months for patients with amyloidosis in contrast to 38.3 months for non-amyloidosis cohort. The median wait time on dialysis for those with kidney failure due to T1DM, T2DM, glomerular disease and PKD was 34.2 months, 37.5 months, 24.2 months and 33.1 months respectively.

**Table 3:** Baseline characteristics of kidney transplants in ANZDATA registry from 1990 and 2022 group by cause of kidney disease

Transplant cohort characteristics	Propensity match for kidney failure vs. amyloidosis									
	Amyloidosis (*n* = 48)	Non-Amyloidosis (*n* = 23,415)	Type 1 Diabetes (*n* = 192)	SMD	Type 2 Diabetes (*n* = 192)	SMD	PKD (*n* = 192)	SMD	Glomerular Disease (*n* = 192)	SMD
Sex										
Male	36 (75)	14,665 (63)	141 (73)	0.06	132 (69)	0.04	128 (67)	0.07	132 (69)	0.01
Female	12 (25)	8,750 (37)	51 (27)		60 (31)		64 (33)		60 (31)	
Transplant Era										
1990–2000	8 (17)	6,755 (29)	28 (15)	0.04	41 (21)	0.07	25 (13)	0.03	28 (15)	0.04
2001–2010	10 (21)	7,484 (32)	52 (27)		34 (18)		38 (20)		40 (21)	
2011–2022	30 (63)	9,176 (39)	115 (60)		120 (63)		129 (67)		124 (65)	
MOT	5 (10)	1,164 (5)	46 (24)	N/A	1 (1)	N/A	5 (3)	N/A	0 (0)	N/A
Donor Type				0.076		0.083		0.016		0.032
Living Donor	4 (8)	2,684 (11)	24 (13)		7 (4)		15 (8)		23 (12)	
Deceased Donor	44 (92)	20,731 (89)	168 (87)		185 (96)		177 (92)		169 (88)	
Age at transplant (years)	55.2 +/-11.1	48.5 +/- 13.2	51.9+/- 13.2	0.092	55.6 +/-12.2	0.02	57.1 +/- 11.7	0.095	55.5 +/- 12.7	0.037
Dialysis time (months)	28.4 +/- 22.7	38.3 +/- 46.9	33.3 +/- 30.5	0.071	33.4 +/-24.1	0.063	37.5 +/- 31.4	0.088	34.3 +/- 33.1	0.074
Smoking History	20 (42)	11,950 (51)	84 (44)	0.012	86 (45)	0.015	70 (36)	0.024	75 (39)	0.034
CLD	4 (8)	1,224 (5)	14 (7)	0.041	16 (8)	0.019	13 (7)	0.08	12 (6)	0.018
CAD	10 (21)	2,587 (11)	63 (33)	0.084	61 (32)	0.045	28 (15)	0.012	31 (16)	0.025
PVD	8 (17)	5385 (23)	65 (34)	0.089	48 (25)	0.067	5 (3)	0.092	9 (5)	0.081
Diabetes	1 (2)	4,899 (21)	192 (100)	N/A	192 (100)	N/A	26 (14)	N/A	43 (22)	N/A
Induction Immunosuppression				N/A		N/A		N/A		N/A
Anti-CD25	32 (67)	15,022 (64)	100 (52)		150 (78)		137 (71)		142 (74)	
OKT3	3 (6)	985 (4)	5 (3)		9 (5)		15 (8)		14 (7)	
Polyclonal Anti-T-cell	1 (2)	2,140 (9)	35 (18)		12 (6)		4 (2)		19 (10)	
Maintenance Immunosuppression				N/A		N/A		N/A		N/A
Prednisone	38 (79)	20,770 (89)	188 (98)		180 (94)		174 (90)		184 (96)	
Tacrolimus	28 (58)	15,449 (66)	140 (73)		125 (65)		132 (69)		123 (64)	
Cyclosporine	9 (19)	8,604 (37)	48 (25)		47 (25)		53 (28)		65 (34)	
Mycophenolate	28 (58)	20,914 (89)	170 (89)		175 (91)		169 (88)		163 (85)	
Azathioprine	5 (10)	4,667 (20)	15 (8)		21 (11)		27 (14)		22 (12)	
mTOR inhibitor	4 (8)	2,549 (11)	8 (4)		11 (6)		14 (7)		10 (5)	

Expressed as n (%) for categorical variables or mean (standard deviation) for continuous variables. Standardised mean differences (SMD) compare amyloidosis with each propensity score-matched comparator cohort. Absolute SMD values < 0.10 were considered indicative of acceptable covariate balance. N/A indicates variables not included in propensity score matching. Immunosuppression variables were reported descriptively only and were not included in propensity score matching because of missingness and lack of dosing or therapeutic drug-level data. For categorical variables with more than two levels, the SMD shown represents the maximum absolute SMD across categories. *Smoking history includes current and former smokers. CLD Chronic Lung Disease. CAD Coronary Artery Disease. PVD Peripheral Vascular Disease. MOT Multi-Organ Transplants

**Table 4:** Sensitivity analyses: Cox and competing-risk models comparing (**A**) survival and (**B**) all-cause graft survival in transplant patients by cause of kidney failure, using amyloidosis as the reference group

Model	T1DM		T2DM		Glomerular disease		PKD	
		*p*-value		*p*-value		*p*-value		*p*-value
**A**								
Multivariable (cs-HR)	0.72 (0.52–0.98)	0.04	1.02 (0.75–1.35)	0.68	0.40 (0.28–0.57)	< 0.001	0.38 (0.29–0.56)	< 0.001
Competing Risks* (sHR)	0.78 (0.65–0.91)	0.035	1.05 (0.77–1.31)	0.45	0.47 (0.38–0.61)	< 0.001	0.42 (0.31–0.55)	< 0.001
**B**								
Multivariable (cs-HR)	0.71 (0.46–1.10)	0.41	1.18 (0.76–1.85)	0.38	0.62 (0.40–0.95)	0.03	0.54 (0.35–0.84)	0.03
Competing Risks* (sHR)	0.87 (0.61–1.14)	0.45	1.22 (0.84–1.47)	0.31	0.89 (0.80–1.09)	0.19	0.68 (0.59–0.91)	0.03

Data is expressed as cause-specific hazard ratio (cs-HR) or subhazard ratio (sHR) and 95% confidence interval (CI). Adjusted for sex, transplant era, age at transplant, time on dialysis, smoking history, chronic lung disease, coronary artery disease and peripheral vascular disease. Reference is Amyloidosis. *Fine and Gray model, competing events of graft loss (return to dialysis). Amyloidosis is the reference group. HRs < 1 indicate lower hazard in the comparator group relative to amyloidosis

#### Survival

**Fig. 4:**
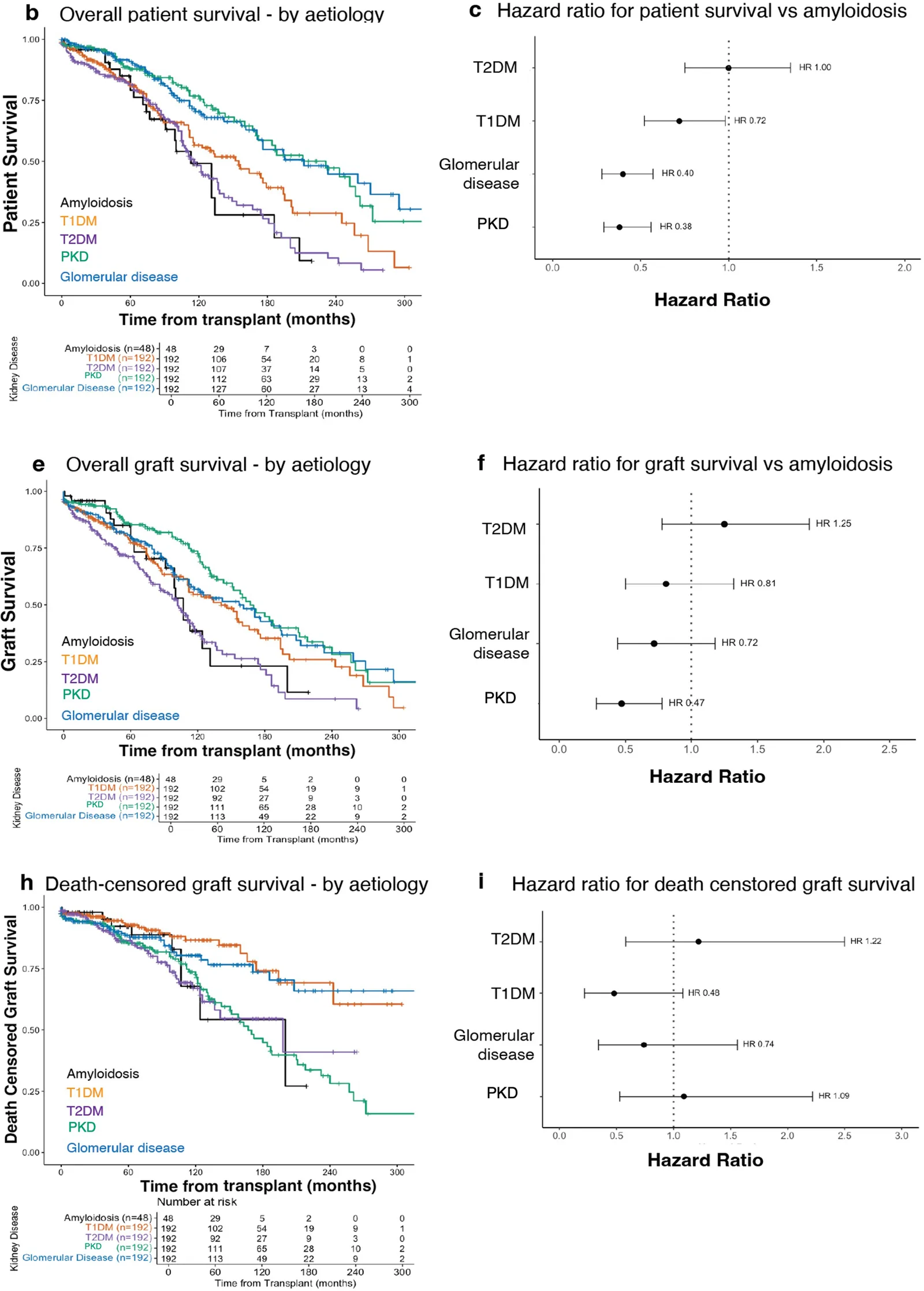
Kaplan Meier survival curves comparing (**A**) patient survival for amyloidosis versus T1DM, T2DM, glomerular disease and PKD amyloidosis, (**C**) all-cause graft survival for amyloidosis versus T1DM, T2DM, glomerular disease and PKD and (**E**) death-censored graft survival for amyloidosis versus T1DM, T2DM, glomerular disease and PKD. Hazard ratios for the specified outcomes (**B**) patient survival post-transplant compared with amyloidosis, (**D**) all-cause graft survival compared with amyloidosis and (**F**) death-censored graft survival compare with amyloidosis. Amyloidosis is the reference group. HRs < 1 indicate lower hazard in the comparator group relative to amyloidosis

The median survival post-transplant was 113 months (95% CI: 91–140 months). Patient survival was worse in patients with amyloidosis compared with kidney failure due to T1DM (HR, 95% CI) (0.72, 0.52–0.98), glomerular disease (0.40, 0.28–0.57), and PKD (0.38, 0.29–0.56), but similar to patients with kidney failure due to T2DM (1.00, 0.75–1.35) (Fig. 4A, B).

The causes of death in transplant recipients with amyloidosis are reported in Table S4. Cardiovascular disease remains the most common cause of death, similar to the non-amyloidosis population, however it should be noted that patients with cardiac involvement of amyloidosis often pass away due to heart failure and its complication rather than coronary artery disease.

Sensitivity analyses showed similar results with survival in patients with amyloidosis comparable to the whole cohorts of those with kidney failure due to T2DM (HR, 95% CI) (1.02, 0.75–1.35) but worse than those with T1DM (0.72, 0.52–0.98), glomerular disease (0.40, 0.28–0.57) and PKD (0.38, 0.29–0.56). Results were similar in the Fine and Gray model (Table 4A). Some evidence of non-proportionality was observed, likely reflecting changes in relative mortality hazards over the long study period and the high early mortality risk in amyloidosis. No major violation was identified that changed the direction of the primary disease-group associations; Cox hazard ratios were therefore interpreted as average associations over follow-up.

Survival analyses comparing transplant recipients with kidney failure due to amyloidosis and the rest of the transplant cohort are shown in Figure S3 and Table S6.

#### All-cause graft survival

The median graft survival for transplant recipients with amyloidosis was 100 months (95% CI: 83–140 months). When compared with matched cohorts, kidney graft survival for patients with amyloidosis was worse than those with PKD (HR, 95% CI) (0.47, 0.28–0.78), but similar to patients with kidney failure due to T2DM (1.25, 0.78–1.89); T1DM (0.81, 0.50–1.32), or glomerular disease (0.72, 0.44–1.18) (Fig. 4C, D).

Sensitivity analyses showed that, on a whole cohort level, graft survival is comparable between transplant recipients with amyloidosis and kidney failure due to T2DM (HR, 95% CI) (1.18, 0.76–1.85) and T1DM (0.71, 0.46–1.10) but worse when compared with glomerular disease (0.62, 0.40–0.95) and PKD (0.54, 0.35–0.84). In Fine–Gray competing-risk regression accounting for death as a competing event, the subdistribution hazard of all-cause graft survival was significantly lower in recipients with PKD (sHR, 95% CI) (0.68, 0.59–0.91), while no significant differences were observed for T1DM (0.87, 0.61–1.14), T2DM (1.22, 0.84–1.47), or glomerular disease (0.89, 0.80–1.09), compared with amyloidosis (Table 4B).

#### Death-censored graft survival

The median death-censored graft survival was 195 months (95% CI 135–243 months). Death-censored graft survival after transplantation for amyloidosis patients was comparable to those with kidney failure due to T2DM (HR, 95% CI) (1.22, 0.58–2.50), T1DM (0.48, 0.22–1.08), glomerular disease (0.74, 0.34–1.56) and PKD (1.09, 0.53–2.22) (Fig. 4E, F). Sensitivity analyses, on a whole cohort level, showed that death-censored graft survival is comparable between patients with amyloidosis and T2DM (HR, 95% CI) (0.71, 0.36–1.41) and T1DM (0.54, 0.27–1.06) but worse when compared with glomerular disease (0.38, 0.19–0.74) and PKD (0.32, 0.16–0.63).

#### Disease recurrence

Amyloidosis recurrence post kidney transplantation is reported qualitatively due to only seven transplant recipients with amyloidosis having recorded disease recurrence. The median time to recurrence being 48 months (IQR: 11–76 months). In two patients, disease recurrence and graft failure occurred at the same time.

### Westmead amyloidosis cohort

A small single-centre Westmead cohort is described in the Supplementary Appendix to provide exploratory contemporary context regarding amyloid subtype distribution; it was not used for inferential comparison or to support the primary registry conclusions.

## Discussion

This contemporary ANZDATA registry study showed that patients with amyloidosis had worse survival on dialysis and post-transplant compared with most other causes of kidney failure and constitutes an intrinsically high-risk condition within this population [4, 25]. Graft survival post-transplant was similar for patients with amyloidosis compared with other causes of kidney failure when death is accounted for. Transplant outcomes should be interpreted cautiously though given a limited number of patients.

The median survival on dialysis for amyloidosis patients was 27 months and significantly shorter than matched cohorts of those with diabetic nephropathy, glomerular disease and PKD. This high risk of mortality is congruent with existing retrospective studies assessing AA and AL amyloidosis [5, 26]. Despite 284 (42.5%) of our patients entering the registry after 2010, these results are similar to the 2013 ANZDATA study of 490 patients between May 1963 and December 2010 [2]. This highlights the disappointing lack of progress in the management of dialysis patients with renal amyloidosis. Importantly, this study shows that patients with amyloidosis exhibited markedly poorer survival than those with diabetic nephropathy, a group typically known to have worse dialysis outcomes owing to systemic cardiovascular burden [21]. Our data did not have sufficient patient-level information to further define whether this is related to extra-renal disease burden, disease control or treatment related toxicities, which is particularly important for patients with AL amyloidosis [4, 5]. Collectively, these data underscore the poor prognosis and complex management challenges faced by patients with amyloidosis on dialysis.

Patients with amyloidosis accounted for only 0.2% of all transplant recipients in this ANZDATA cohort. Currently, guidelines recommendations for patients with AL amyloidosis, in particular, is that they should have minimal extra-renal disease, have received a potentially curative regimen and be in stable remission prior to consideration for transplantation [27]. When combined with the high risk of disease recurrence and prognostic uncertainty of patients with all causes of systemic amyloidosis, it is unsurprising that few undergo transplantation [28]. Post-transplant survival in this group reached a median of 113 months, which is better than survival for the group included in the earlier ANZDATA analysis (median: 72 months) [2]. The discrepancy between all-cause mortality relative to the previous ANZDATA analysis may indicate enhanced recognition and selection of appropriate transplant candidates, consistent with the fact that the majority of our cohort (62.5%) received their kidney transplant after 2010. Median patient survival in our amyloidosis cohort is similar to post-transplant survival in other retrospective studies for patients with AL amyloidosis [6–8]. Only limited comparisons can be made with retrospective AA amyloidosis cohorts reported in the literature [14, 29], due to study heterogeneity.

Our study found that post-transplant outcomes were similar between patients with kidney failure due to T2DM and amyloidosis, which is consistent with findings from other international registry based studies [12, 30]. Given that T2DM is the most common indication for kidney transplantation in Australia and New Zealand, these findings suggest that, in carefully selected patients with kidney failure attributed to amyloidosis, transplantation may offer acceptable outcomes. Interpretation is limited by residual confounding, lack of amyloid subtype data, and likely selection bias.

The median all-cause graft survival in transplant recipients with amyloidosis was 100 months, similar to existing AL amyloidosis cohorts [6, 7, 9]. Death-censored graft survival did not differ significantly between patients with amyloidosis and those with other causes of kidney failure. By delineating mortality risk and all-cause graft survival, our findings indicate that patient mortality, rather than immunologic or technical graft failure, is the principal determinant of graft loss, reflecting the systemic nature of amyloidosis and consistent with observations from a recent U.S. registry analysis [30]. Overall, these findings reinforce the feasibility and benefits of transplantation in selected amyloidosis patients.

Our study included a multinational, population-based cohort with comprehensive longitudinal data spanning three decades, enabling robust multivariable and matched analyses. Additionally, universal health coverage in Australia and New Zealand mitigates disparities in healthcare access that might confound outcome comparisons in other settings. The major limitation of this study is the lack of subtype classification in registry data to directly compare between the types of amyloidosis, as each subtype is a discrete disease with a different renal prognosis and treatment. Additionally, individual patient level data was not available to assess treatment era and changes in care, which would have been a more useful covariate rather than KRT decade, especially with new daratumumab-based treatment regimens arising for AL amyloidosis that have become highly effective in allowing for organ recovery and a long duration of remission [10]. As such, a comparison of survival based on KRT decade is vulnerable to misclassification if thought to be related to newer therapies. These reflect general limitations of registry analyses. As a result, residual confounding by unmeasured factors such as hematologic response, cardiac involvement, treatment data and frailty remains. These factors are major determinants of dialysis survival, transplant candidacy and post-transplant mortality. Therefore, the observed associations should not be interpreted as subtype-specific causal effects. This limitation is particularly important for the transplant cohort, where selection for transplantation is likely to be highly non-random and influenced by disease control, cardiac status and clinician assessment of prognosis.

## Conclusion

Patients with kidney failure attributed to amyloidosis experience markedly reduced survival on dialysis compared with other major causes of kidney failure. Among kidney transplant recipients, amyloidosis was associated with less favourable post-transplant patient survival than several comparator groups, whereas death-censored graft survival appeared broadly comparable. These findings suggest that transplantation may offer acceptable graft outcomes in carefully selected patients, but interpretation is limited by amyloidosis subtype heterogeneity, residual confounding, absence of cardiac and haematologic response data, and likely selection bias in transplant candidacy. Future registry and cohort studies should incorporate amyloidosis subtype, treatment response, cardiac involvement and contemporary amyloid-directed therapies to better define prognosis and guide transplant selection.

## Supplementary Information

Below is the link to the electronic supplementary material.

Supplementary Material 1

## Data Availability

The datasets analysed during the current study are available upon request from the ANZDATA registries; https://anzorrg.org.au/registries/anzdata.

## Abbreviations

PKD: Polycystic kidney disease
ANZDATA: Australia and New Zealand Dialysis and Transplant Registry
KRT: Kidney replacement therapy
T1DM: Type 1 Diabetes
T2DM: Type 2 Diabetes
PSM: Propensity score matching
csHR: Cause-specific hazard ratio
SMD: Standardised mean difference
sHR: Subdistribution hazard ratio
