# Supplementary material for: Outcomes following dialysis and transplantation in amyloidosis: ANZDATA analysis
Source: BMC Nephrol. 2026 Aug 3;27:642. doi: 10.1186/s12882-026-05230-3 (PMC13726128; doi:10.1186/s12882-026-05230-3)
Supplement: Supplementary file 1 [file 12882_2026_5230_MOESM1_ESM.docx]

**Supplementary Data**

Supplementary Exploratory Cohort: Westmead Amyloidosis Cohort

Given the small number of patients requiring dialysis or transplantation within individual amyloid subtypes, these subtype-specific survival estimates are descriptive only and were not used to support the primary conclusions of the ANZDATA registry analysis.

Amyloid subtype specific outcomes: A retrospective, single-centre cohort review was performed of patients reviewed through the Westmead amyloidosis clinic between the period 01/01/2020 to 01/06/2025, and patients who received a kidney or kidney-pancreas transplant at Westmead Hospital anytime between 01/01/1990 to 01/01/2025. Median survival on dialysis and post-transplant were determined using Kaplan-Meier survival analyses. Patients were documented as having kidney involvement if there was biopsy proven disease or the presence of proteinuria with no other predisposing clinical factors as judged by a nephrologist.

Westmead Amyloidosis Clinic Summary

A total of 252 systemic amyloidosis patients were reviewed in the Westmead amyloidosis clinic between 2020–2025 (**Table S7**). Eight patients required dialysis (n (AL) = 5, n (AA) = 2, n (AFib) = 1) during a median follow up time of 43 months. Dialysis was required at a median time of 21.1 months (95% CI: 1 - 45.4 months) after amyloidosis diagnosis. Median survival on dialysis was 66.4 months (95% CI: 37-83.5 months), with the median survivals for AA and AL amyloidosis being 22 and 74 months respectively. Six additional patients underwent kidney transplantation [AL (n =1), AA (n=4), AFib (n=1)] at a mean age of 60.8 years. The median time between diagnosis of amyloidosis and transplantation was 57.6 months (95% CI: 28 - 71 months), with the median time on dialysis prior to transplant being 28 months (95% CI: 15 – 60 months). The median survival post-transplant was 64 months (95% CI: 26.8 – 80.4 months), with the median survival for AA amyloidosis being 54 months. The patients’ with AFib and AL amyloidosis lived for 44 and 100 months, respectively. All causes of graft failure were due to patient death. Out of 2590 patients who received a kidney transplant at Westmead Hospital between 1990 – 2025, we identified 5 patients who developed de novo amyloidosis [AL (n=4), ATTRwt (n=1)] at a median time of 102 months post kidney transplant. The median survival post-amyloidosis diagnosis was 26 months (95% CI: 8.4-42.4 months).

**Supplementary tables**

**Table S1: Studies of patients with amyloidosis patients needing kidney replacement therapy**

| **Amyloid type** | **Study Design** | **Results** | | |
| --- | --- | --- | --- | --- |
| **Combined** |  | Patient survival | Allograft survival | Disease recurrence |
| Ng et al,^1^  N = 286 | Retrospective cohort study of kidney transplant recipients with plasma cell dyscrasias and amyloidosis between 2006 to 2018 using the UNOS registry  Median follow up: 4.85 years | Higher risk of death with amyloidosis  (aHR 2.43, 95% CI 1.75-3.38) | Higher rate of overall graft loss in amyloidosis vs other diseases  (aHR 1.98, 95% CI 1.46-2.68) | N/A |
| Sawinski et al,^2^  N = 576 | Retrospective propensity scores matched cohort study using data from the UNOS registry of transplanted patients between 1987 to 2015.  Median follow up: Follow-up till March 2016 | Median survival of 5.8 years.  The 1- and 5-year survival rates of 91% and 70% post transplantation | Median graft survival of 4.8 years | Graft loss due to recurrent disease in 15.7% of cases |
| Tang et al^3^  N = 490 | Retrospective ANZDATA analysis for patients on dialysis or transplanted between 1963-2010.  Median follow up: Follow up till December 2010 | The median survival on dialysis was 2.09 years for patients with amyloidosis compared to 4.45 years for other causes of kidney failure.  The median survival of post kidney transplantation was 6.03 years for patients with amyloidosis, compared to 16.8 years for other causes of kidney failure | Median first graft survival was 4.55 years compared to 10.7 years for other causes of kidney failure. | Nine allografts (16.4%) experienced recurrence of original amyloidosis compared to 3% for other causes of kidney failure. |
| Yamauchi et al^4^  n = 643 | Retrospective US OPTN kidney transplant registry analysis for the period between 2002-2021.  Median follow up: 5 years | Post-transplant 5-year patient survival rate was 79.8% (similar rate to diabetic nephropathy) | Post-transplant 5-year death-censored graft survival rate was 88.6% | N/A |
| **AL Amyloidosis** | | | | |
| Angel-Korman et al^5^  n = 49 | Retrospective cohort study of kidney transplantation at the Amyloidosis Center at Boston University between 1987-2017  Median follow up: 7.2 years | Post-transplant median survival was 10.5 years | Post-transplant median graft survival was 10.4 years.  One, three, and five-year graft survival were 94%, 89%, and 81%, respectively. | Fourteen patients (29%) had recurrence amyloidosis in the graft with median to recurrence being 3.7 years. |
| Bergesio et al,^6^  N=59 | Retrospective multi-centre cohort study across Italy of with biopsy-proven systemic amyloidosis between January 1995 and December 2000 and required dialysis  Median follow up: 25 years | Median survival on dialysis was 11 months | N/A | N/A |
| Bollee et al ^7^  n = 19 | Multi-centre retrospective cohort from five dialysis units in France between 1995-2005.  Median follow up: 37.4 months | Median survival on dialysis was 26 months with high rate of extrarenal involvement, especially cardiac disease (63%) in this cohort. | N/A | N/A |
| Cohen et al^8^  n = 50 | Retrospective case series of kidney transplantation from the UK National Amyloidosis Database 2005 – 2020  Median follow up: 8.9 years | 13/50 (32.5%) of patients died | Median graft survival was 12.4 years (95% CI 10.7–14.2 years). | Three patients had proven amyloidosis recurrence in the renal allograft |
| Decourt et al^9^  n=267 | Registry analysis of all patients who had kidney failure between January 1, 2002 and December 31, 2011 in France and required dialysis.  Median follow up: 27 months | Median survival was 28.9 months | N/A | N/A |
| Gertz et al^10^  n = 38 | Retrospective cohort study with patients diagnosed with AL amyloidosis at Mayo clinic between 1993 to 1997.  Median follow up: 11 years | Median survival on dialysis was 10.4 months. | N/A | N/A |
| Gertz et al,  N=37^11^ | Retrospective cohort study of 37 dialysis patients with AL amyloidosis managed at Mayo clinic between 1981 to 1990.  Median follow up: 11 years | The median survival from initiation of dialysis 8.2 months. The 10 patients who received peritoneal dialysis survived for a median of 12.2 months and the 27 who received haemodialysis survived for 7.7 months, which was not significantly different. | N/A | N/A |
| Havasi et al^12^  n = 237 | Multi-national retrospective study of kidney transplants from 1987-2020  Median follow up: 8.5 years | Post-transplant median overall survival was 8.6 years. One-, three- and five-year OS from renal transplantation was 95%, 83% and 74%, respectively Median follow up was 8.5 years post-transplant. | median graft survival was 7.8 years. One-, three- and five-year graft survival rates were 92%, 79% and 69%, respectively. | Sixty-nine patients (29%) experienced hematologic relapse. Median time to recurrence was 6.6 years. |
| Herrmann et al^13^  n = 19 | Retrospective case series of kidney transplantation for AL amyloidosis at the Mayo clinic between 1999 to 2008.  Median follow up: 41.4 months | 79% of patients were alive over median follow up time of 41.4 months | All graft losses were due to patient death | 2 out of the 19 patients had recurrent disease (10.5%). |
| Heybeli et al^14^  n = 60 | Retrospective cohort study of kidney transplantation for AL amyloidosis at Mayo Clinic between 1997 and 2018  Median follow up: 61 months | median overall survival post-transplant was 123 months. Median follow up time 61 months. | 1- and 5- year post transplant death censored graft survival was 98.3 and 95.8% respectively | The median time to amyloid recurrence  was 181 vs 81 months in those who achieved CR compared to VGPR |
| Law et al^15^  n = 51 | Retrospective cohort study of kidney transplantation at the UK National Amyloidosis Centre 1989-2018.  Median follow up: 6.1 years | 1-, 5- and 10- year patient survival was 96%, 79% and 39% respectively. | One-, 5- and 10-year death-censored unadjusted graft survival was 98, 93 and 93% respectively. | Recurrent amyloid in the allograft occurred in 14% |
| Leung et al^16^  n=72 | Retrospective cohort study of all patients with AL amyloidosis who received a stem cell transplant between March 1996 and January 2010 in USA who required dialysis either before or after stem cell transplant  Median follow up: 52 months | The median survival of all patients who required dialysis was 48.2 months but in those who did not have renal recovery, the median survival was 30 months. | N/A | N/A |
| Pinney et al^17^  n = 25 | Retrospective cohort study of kidney transplantation the UK Amyloidosis Centre between 1978-2012.  Median follow up: 4.6 years | 13/25 (52%) had died by time of censoring. | Median graft survival of 5.8 years | 7/25 (28%) had recurrent amyloid in the allograft |
| Sattianayagam et al^18^  n = 22 | Retrospective cohort of kidney transplantation for AL amyloidosis from UK Amyloidosis Centre 1984-2009.  Median follow up: 4.8 years | 1- and 5-year patient survival was 95% and 67% respectively. Median survival post-transplant was 6.5 years. | 2 out of the 22 patients had graft failure at end of study follow up (9.1%) | no renal graft failed due to recurrent amyloid during follow up period. |
| **AA amyloidosis** |  |  |  |  |
| Abedi et al,^19^  N=18 | Retrospective cohort study of patients with FMF-related AA amyloidosis who needed a kidney transplant till 2013 at Imam Reza Hospital, Iran  Median follow up: minimum 5 years | The 1 and 5- year survival rates were 100% and 88.8%. | The 1 and 5- year graft survival rates were 94.7% and 94.4%. | One patient had disease recurrence |
| Altindal et al,^20^  N=24 | Retrospective cohort study of 24 kidney transplant recipients with AA Amyloidosis (FMF) between 1991 and 2012 at a single centre in Turkey  Median follow up: 80.3 months | 5- and 10- year patient survival rates were 87.5% and 65.6%. | 5- and 10- year death censored graft survival rates were 95.8% and 78.4%. | Two patients had disease recurrence |
| Bergesio et al,^6^  N=37 | Retrospective multi-centre cohort study across Italy of 373 patietns with biopsy-proven systemic amyloidosis between January 1995 and December 2000. 37 of the patients with AA amyloidosis ended up on dialysis.  Median follow up: 28 months | Median survival on dialysis was 17 months | N/A | N/A |
| Bitik et al^21^,  n=31 | Retrospective cohort study of 31 patients who underwent kidney transplantation for AA amyloidosis secondary to FMF between 1994 and 2021 at Baskent University Hospital (Ankara, Turkey)  Median follow up: 108 months | 84% and 48% at 5 and 10 years. | Graft survival was 71% and 45% at 5 and 10 years. | 3/31 (9.7%) had disease recurrence during the study |
| Bollee et al^7^  n = 20 | Multi-centre retrospective cohort study of patients in France between 1995-2005.  Median follow up: 31.8 months | Survival on dialysis ranged from 29 to 113 months | N/A | N/A |
| Emiroglu et al, ^22^  N=32 (n=22 FMF, n=1 rheumatoid arthritis, n=9 idiopathic) | Retrospective cohort study of 32 patients with AA amyloidosis who received a kidney transplant between 1985 and July 2004  Median follow up: 51.2 months | 1- and 5-year patient survival rates were 90.6% and 84.3% | 1- and 5- year graft survival rates were 81.2% and 68.7%. | Two patients had disease recurrence. |
| Gursu et al^23^  n = 44 | Multi-centre retrospective cohort study of 44 kidney transplant in Turkey from 1983 to 2009  Median follow up: 8 years | 1, 5, and 10 years were 87.6%, 78.1%, and 62.3% | 1, 5 and 10 years were 87.6%, 75.4%, 56.4% | 4/44 had recurrent disease (9.1%) |
| Haq et al^24^  N=13 | Retrospective cohort study of 13 kidney transplant recipients between 1985 to 2001 in Ireland (n=11 rheumatoid arthritis, n=1 IBD, n=1 idiopathic)  Median follow up: 5 years | 1 and 5-year patient survival rates were 69% and 69%. | 1- and 5- graft survival rates were 56% and 56%. | N/A |
| Keven et al,^25^  N=17 (FMF) | Retrospective cohort study of 17 kidney transplant recipients at Ankara University school of medicine between 1978 and 2003.  Median follow up: 63 months | 1- and 5- patient survival rates were 94% and 90%. | 1- and 5- year graft survival rates were 93% and 89%. | Two (12%) had recurrence in the transplanted kidney |
| Koc et al,^26^  n=29 | Retrospective cohort study of dialysis patients with AA amyloidosis on peritoneal dialysis at a single centre in Turkey between 2001 to 2011  Median follow up: 9.5 years | The mortality rate of patients with amyloidosis was much higher than that of other causes of kidney failure, especially in the first 2-3 years (p<0.001), suggestive loss of PD survival advantage | N/A | N/A |
| Kofman et al^27^  n = 59 (n=25 inflammatory disease, n=21 FMF, n=10 chronic infection) | Multi-centre retrospective cohort study in France for transplant recipients between 1981 to 2009  Median follow up: 5.5 years | 5- and 10-year patient survival was 82.5% and 61.7%, which was significantly lower than other causes of kidney failure (94.2% and 83.4% respectively). | 5- and 10- year death-censored graft survival was 93.5% and 77.5%, which did not differ significantly from other causes of kidney failure. | Eight (14%) patients had recurrence of AA amyloidosis |
| Kuroda et al, ^28^  N=51 | Retrospective cohort study of 51 patients with kidney failure due to AA amyloidosis (rheumatoid arhtirits) on haemodialysis between 1982 and 2005 at Niigata University Hospital  Median follow up: 96.9 person years | Survival of these 51 patients from the initiation of HD at 38 days was 75%, at 251 days was 50%, and at 1,483 days was 25%. | N/A | N/A |
| Law et al^15^  n = 48 | Retrospective cohort study of kidney transplant at the UK National Amyloidosis Centre in 1989-2018.  Median follow up: 6.1 years | 1-, 5- and 10-year survival 96, 84, 66% respectively | One-, 5- and 10-year death-censored unadjusted graft survival was, respectively, 94, 91 and 78%. | Recurrent amyloid occurred in renal allograft in 9/48 (19%). |
| Ozdemir et al, ^29^  N=55 | Retrospective cohort study of 55 patients with kidney failure due to FMF AA amyloidosis at a single centre in Turkey till 2005. 25 patients received a kidney transplant and 30 patients were on haemodialysis.  Median follow up: 68.8 months | The 1- and 2- and 5- year survival rates for the transplant patients were 86.9%, 82.6% and 78.2%.  The 1- and 2- and 5- year survival rates for the haemodialysis patients were 60.7%, 50% and 46.4%.  The median survival with transplant was 50.3 months and 33.3 months on haemodialysis. | N/A | N/A |
| Ozdemir et al, ^30^  N=30 | Retrospective cohort study of 30 kidney transplant recipients with AA amyloidosis due to FMF (70%), idiopathic (10%), tuberculosis (10%) and rheumatoid arthritis (10%) at a single centre in Turkey between 1985 to 2001  Median follow up: 68 months |  |  | 20 patients (66.7%) developed a recurrence of amyloidosis in their allografts, with time to diagnosis from 18 months to 10 years. |
| Pinney et al^17^  n = 43 (n=16 inflammatory arthritis, n=10 FMF, n=9 IBD, n=5 recurrent infection, n=2 Castleman’s, n=2 undefined) | Retrospective cohort study of all patients from the UK Amyloidosis Centre who received a kidney transplant between 1978-2012.  Median follow up: 5.1 years | Sixteen (37.2%) patients died by time of censoring | Median graft survival was 10.3 years. Eight (18.6%) had failed at censoring | 9/43 (19.5%) had recurrent AA amyloidosis post-transplant. |
| Sahutoglu et al,^31^  n=41 | Retrospective cohort study of 41 patients with AA amyloidosis who underwent kidney transplantation between 1988 and 2012 at the Istanbul faculty of medicine.  Median follow up: 70.9 months | Median patient survival was 185.5 months. | Median graft survival was 232.2 months. | 50% of patients with AA amyloidosis had recurrence of proteinuria, presumed to be disease recurrence. |
| Sarihan et al^32^  n = 81 (FMF) | Multi-centre retrospective analysis of four transplant centres in Turkey from first transplant till 2019  Median follow up: 90 months | 5- and 10-year survival 92.5% and 70.4% respectively | 5- and 10-year graft survival rates were 94.7% and 53.8% in those with recurrent disease whilst it was 95%, and 77.8% in those without recurrent disease. | AA Amyloidosis recurred in 21 of 81 patients. |
| Saritas et al, ^33^  N=27 (FMF) | Retrospective cohort study of 27 kidney transplant recipients between 2005 and 2018 at Ankara university medical school.  Median follow up: 6.5 years | 1,5 and 10-year patient survival rates were 100%, 95.8% and 95.6%. | 1,5 and 10- year graft survival rates were 96.2%, 96.2%, 96.2%. | N/A |
| Schwarz et al^34^  n = 86 | Multi-centre retrospective study of kidney transplant recipients between 2008-2018 across 26 centres in France.  Median follow up: 55.8 months | 1-, and 5-year survival rates were 85.5% and 84% | 1- and 5- year graft loss rates was 10.5% and 13% respectively | Disease recurrence occurred in 5/86 (5.8%) of patients. |
| Sherif et al^35^  n = 23 (n=16 FMF, n=7 idiopathic) | Multi-centre cohort study of 23 kidney transplant at in Egypt between 1984-2000  Median follow up: 82 months | 5- and 10-year patient survival rates were 80% and 68% | 5- and 10-year graft survival rates were 79.35% and 65.92% respectively | Recurrence of disease occurred in 1//23 patients (4.3%). |
| Tatar et al, ^36^  N=32 | Retrospective cohort study of 32 patients with FMF related AA amyloidosis who received a kidney transplant between 1999 and 2015 at a single centre in Turkey  Median follow up: 61 months | 18.7% of patients died during the follow up period. | 9.3% of patients has graft failure and a return to haemodialysis. | N/A |
| Ylinen et al^37^  n = 37 | Retrospective cohort study of patients at Helsinki University Central Hospital between 1974 and 1987.  Median follow up: 54 months | on dialysis, the 1-, 2- and 3- year survival was 82%, 46% and 37% respectively. Post kidney transplant, the 1-, 2- and 3- year survival 70%, 62%, and 62% respectively | N/A | N/A |
| **Hereditary amyloidosis** | |  |  |  |
| Gillmore et al^38^  n = 10 AApoA1 | Multi-centre cohort study of transplant recipients in the UK needing transplant between 1978- 2005.  Median follow up: 9 years | Eight of 10 patients were alive at follow up (80%). | Seven out of 10 patients had a functioning graft at follow up (70%) | Five out of 10 patients had disease recurrence (50%). |
| Gillmore et al, 2009^39^  n = 44 AFib | Cohort study of patients with fibrinogen amyloidosis at the UK National Amyloidosis Centre between 1992 to 2007. 44 patients had kidney failure who needed dialysis and 10 of these underwent kidney transplantation.  Median follow up: 4 years | Median survival from commencement of dialysis was 9.3 years when censored for transplantation. Median survival post-transplant was 5.8 years. | Seven grafts had failed after median follow up from transplantation of 5.8 yr. Median overall graft survival was 5.9 years | 3/10 (30%) had disease recurrence after 5.8, 6.0, and 7.4 years |
| Meyer et al, 2020^40^  n = 19, AFib | Cohort study of fibrinogen amyloidosis patients diagnosed by genetic testing in France between 1983-2014, with 19 undergoing transplantation (n=15 kidney alone, n=4 liver kidney transplant).  Median follow up: 93 months | 10- and 30- year patient survival rates were 84% and 70% respectively. | 5-, 7-, and 9-year kidney graft survival rates were 88%, 80%, and 72%, respectively | Recurrence in 7 kidney-alone transplant (46%) and no recurrence in the liver-kidney transplants |
| Pinney et al, 2013^17^  n = 19 AFib | Retrospective cohort study of fibrinogen amyloidosis attending the UK Amyloidosis Centre who received a kidney transplant between 1978 - 2012. 10 received a kidney-alone and 9 received a liver-kidney transplant  Median follow up: 3.3 years | 7/19 (36.8%) died by time of censoring. | Median graft survival was 7.3 years with kidney-alone and 6.4 years with liver-kidney transplant | Recurrence in 7/10 (70%) of patients with kidney alone. No patients with liver-kidney transplant had disease recurrence. |
| Pinney et al, 2013^17^  n=14 AApoA1 | Retrospective cohort study of apolipoprotein amyloidosis attending the UK Amyloidosis Centre who received a kidney transplant between 1978 and 2012. 12 received a kidney-alone transplant and 2 received a liver-kidney transplant  Median follow up: 9.8 years | 1/14 (7.1%) died by time of censoring | Median kidney graft survival was 13.1 years. | 3/14 (18.8%) had disease recurrence, all of whom had a kidney-alone transplant |
| Pinney et al, 2013^17^  n=3 ALys | Retrospective cohort study of lysozyme amyloidosis attending the UK Amyloidosis Centre who received a kidney transplant between 1978 and 2012.  Median follow up: 2.9 years | All patients were alive at time of censoring | All were functioning at time of censoring | No evidence of disease recurrence |

**Table S2: Details of remaining missing data in the sensitivity analyses.**

A) **Dialysis Cohort**

| **Covariate** | **Diabetic nephropathy n (%)** | **Glomerular disease n (%)** | **PKD n (%)** |
| --- | --- | --- | --- |
| Sex | 265 (0.8) | 184 (1.6) | 65 (1.3) |
| Smoking status | 2467 (8.3) | 1050 (9.0) | 382 (7.6) |
| Coronary artery disease | 1901 (6.4) | 733 (6.3) | 267 (5.3) |
| Chronic lung disease | 1945 (6.6) | 957 (8.2) | 326 (6.5) |
| Peripheral vascular disease | 1802(6.1) | 854 (7.3) | 357 (7.1) |

B) **Transplant Cohort**

| **Covariate** | **T1DM n (%)** | **T2DM n (%)** | **Glomerular disease n (%)** | **PKD n (%)** |
| --- | --- | --- | --- | --- |
| Sex | 12 (0.7) | 19 (0.8) | 36 (0.4) | 21 (0.6) |
| Donor type | 41 (2.6) | 78 (3.5) | 105 (1.3) | 67 (2.1) |
| Smoking status | 132 (8.6) | 205 (9.2) | 657 (7.9) | 235 (7.5) |
| Coronary artery disease | 94 (6.1) | 170 (7.6) | 529 (6.4) | 182 (5.8) |
| Chronic lung disease | 109 (7.1) | 211 (9.5) | 631 (7.6) | 165 (5.3) |
| Peripheral vascular disease | 148 (9.6) | 216 (9.7) | 710 (8.5) | 290 (9.3) |

**Table S3: Cause of death in the Amyloidosis Dialysis Cohort**

| **Cause** | **N (% of all deaths)** |
| --- | --- |
| Cardiovascular disease | 210 (39.1) |
| Infection | 71 (13.2) |
| Malignancy | 36 (6.7) |
| Dialysis withdrawal | 138 (25.7) |
| Other causes – not documented | 82 (15.4) |

**Table S4: Cause of death in the Amyloidosis Transplant Cohort**

| **Cause** | **N (% of all deaths)** |
| --- | --- |
| Cardiovascular disease | 7 (31.8) |
| Infection | 7 (31.8) |
| Malignancy | 2 (9.1) |
| Pulmonary embolus | 1 (4.5) |
| Other causes – not documented | 5 (22.7) |

**Table S5: Cox multivariate regression model demonstrating effect of amyloidosis compared with all other causes of kidney failure on survival of dialysis patients.**

| **Model** | **Univariable** | | **Multivariable** | | **Competing Risks*** | |
| --- | --- | --- | --- | --- | --- | --- |
|  | cs-HR  (95% CI) | p-value | cs-HR  (95% CI) | p-value | sHR (95% CI) | p-value |
| No Amyloidosis | 0.41  (0.34 to 0.48) | <0.001 | 0.45  (0.42 to 0.49) | <0.001 | 0.38  (0.25 to 0.74) | <0.001 |

*Data is expressed as cause-specific hazard ratio (cs-HR) or sub-hazard ratio (sHR) and 95% confidence interval (CI)*

*Adjusted for sex, dialysis era, age at dialysis commencement, dialysis modality, smoking history, chronic lung disease and peripheral vascular disease. Reference is Amyloid. *Fine and Gray model, competing events of transplant.* *Amyloidosis is the reference group. HRs <1 indicate lower hazard in the comparator group relative to amyloidosis.*

**Table S6: Cox multivariate regression model demonstrating effect of amyloidosis compared with all other causes of kidney failure on transplant patient survival**

| **Model** | **Univariable** | | **Multivariable** | | **Competing Risks*** | |
| --- | --- | --- | --- | --- | --- | --- |
|  | cs-HR  (95% CI) | p-value | cs-HR  (95% CI) | p-value | sHR (95% CI) | p-value |
| No Amyloidosis | 0.54 (0.23-0.84) | 0.005 | 0.44 (0.25-0.78) | 0.01 | 0.64  (0.43 to 0.85) | 0.01 |

*Data is expressed as cause-specific hazard ratio (cs-HR) and 95% confidence interval (CI). Adjusted for sex, transplant era, age at transplant, time on dialysis, smoking history, chronic lung disease, coronary artery disease and peripheral vascular disease. Reference is Amyloid*

**Fine and Gray model, competing events of graft loss (return to dialysis).* *Amyloidosis is the reference group. HRs <1 indicate lower hazard in the comparator group relative to amyloidosis.*

**Table S7:** **Summary of the Westmead Amyloidosis Clinic cohort from 2020 – 2025.**

| **Amyloid type** | **N** | **Male Sex**  **(n, %)** | **Age (years)** | **Kidney involvement (%)** | **Cardiac involvement (%)** | **eGFR at diagnosis** | **uACR at diagnosis** | **Dialysis (n, %)** | **Kidney Transplant (n, %)** |
| --- | --- | --- | --- | --- | --- | --- | --- | --- | --- |
| Light chain (AL) | 87 | 53 (60.9) | 63.4 (14.9) | 70.1 | 70.2 | 66.9 (23.7) | 389 (431.2) | 5 (5.7) | 1 (1.1) |
| Amyloid A (AA) | 14 | 10 (71.4) | 59.5 (14.5) | 92.9 | 7.1 | 43 (26.1) | 460 (344.3) | 2 (14.3) | 4 (28.6) |
| Transthyretin (TTRw) | 82 | 64 (78) | 79.2 (12.5) | 0 | 92.6 | 63.6 (19.2) | 15.1 (35.5) | 0 (0) | 0 (0) |
| Transthyretin  (TTRv) | 55 | 31 (55) | 54.9 (16.2) | 3.6 | 41 | 72.1 (13.6) | 11.9 (46) | 0 (0) | 0 (0) |
| Fibrinogen  (AFib) | 8 | 5 (62.5) | 59.2 (9.7) | 100 | 0 | 39.6 (25.6) | 250.3 (203) | 1 (12.5) | 1 (12.5) |
| Lectin  (ALECT2) | 3 | 2 (66.7) | 70.6 (4.5) | 100 | 0 | 28.9 (6.1) | 59.8 (64.4) | 0 (0) | 0 (0) |
| Gelosin (AGel) | 1 | 0 (0) | 34 | 100 | 0 | 55 | 121 | 0 (0) | 0 (0) |
| Apolipoprotein (AApoA1) | 1 | 1 (100) | 46 | 100 | 0 | 19 | 100 | 0 (0) | 0 (0) |
| Apolipoprotein (AApoC2) | 1 | 0 (0) | 76 | 100 | 100 | 22 | 357 | 0 (0) | 0 (0) |

Expressed as n (%) for categorical variables or mean (standard deviation) for continuous variables. eGFR estimated glomerular filtration rate (ml/min/1.73m2), UACR urine albumin to creatinine ratio (mg/mmol) – UACR not available for all patients at time of diagnosis). TTRw wild-type transthyretin amyloidosis, TTRv genetic variant transthyretin amyloidosis

**Table S9:** **Changes in Standardised Mean Differences before and after Propensity-Score Matching** **for Dialysis Patients**

| **Covariate** | **Diabetic nephropathy** | **PKD** | **Glomerular disease** |
| --- | --- | --- | --- |
| Sex | 0.2 to 0.016 | 0.07 to 0.044 | 0.11 to 0.072 |
| Dialysis Era | 0.11 to 0.002 | 0.06 to 0.016 | 0.07 to 0.037 |
| Age at Dialysis | 0.47 to 0.03 | 0.75 to 0.026 | 1.1 to 0.032 |
| Dialysis Modality | 0.02 to 0.002 | 0.09 to 0.063 | 0.15 to 0.09 |
| Smoking History | 0.2 to 0.03 | 0.23 to 0.05 | 0.2 to 0.07 |
| Chronic Lung Disease | 0.19 to 0.09 | 0.29 to 0.046 | 0.16 to 0.056 |
| Coronary Artery Disease | 0.39 to 0.01 | 0.25 to 0.04 | 0.22 to 0.005 |
| Peripheral Vascular disease | 0.69 to 0.04 | 0.19 to 0.009 | 0.14 to 0.07 |

*Numbers in each column represent the standardised mean difference before and after propensity-score matching*

**Table S10: Changes in Standardised Mean Differences before and after Propensity-Score Matching for Transplant Patients**

| **Covariate** | **Type 1 Diabetes** | **Type 2 Diabetes** | **PKD** | **Glomerular Disease** |
| --- | --- | --- | --- | --- |
| Sex | 0.23 to 0.06 | 0.2 to 0.04 | 0.11 to 0.07 | 0.12 to 0.044 |
| Transplant Era | 0.32 to 0.04 | 0.19 to 0.07 | 0.18 to 0.03 | 0.08 to 0.04 |
| Age at Transplant | 0.11 to 0.092 | 0.41 to 0.02 | 0.51 to 0.095 | 0.64 to 0.037 |
| Dialysis Time | 0.32 to 0.071 | 0.28 to 0.063 | 0.21 to 0.088 | 0.17 to 0.074 |
| Donor Type | 0.12 to 0.076 | 0.18 to 0.083 | 0.12 to 0.016 | 0.23 to 0.032 |
| Smoking History | 0.36 to 0.012 | 0.2 to 0.015 | 0.29 to 0.024 | 0.56 to 0.034 |
| Chronic Lung Disease | 0.42 to 0.041 | 0.25 to 0.019 | 0.11 to 0.08 | 0.39 to 0.018 |
| Coronary Artery Disease | 0.21 to 0.084 | 0.37 to 0.045 | 0.17 to 0.012 | 0.27 to 0.025 |
| Peripheral Vascular disease | 0.18 to 0.089 | 0.54 to 0.067 | 0.136 to 0.092 | 0.22 to 0.081 |

*Numbers in each column represent the standardised mean difference before and after propensity-score matching*

**Supplementary Figures:**

**Figure S1: Comparison between different studies demonstrating (A) median survival on dialysis, (B) median survival post- kidney transplant and (C) median all-cause graft survival post-kidney transplant.**

**A)**

**B)**

**C)**

**Figure S2: Kaplan Meier analysis comparing survival of dialysis patients with amyloidosis compared to those without**

**Figure S3: Kaplan Meier survival analyses comparing A) survival, B) all-cause graft survival and C) death-censored graft survival between kidney transplant recipients with amyloidosis to those without**

**C)**

**A)**

**B)**

**Supplementary References**

1.	Ng JH, Izard S, Murakami N, Jhaveri KD, Sharma A, Nair V. Outcomes of kidney transplantation in patients with myeloma and amyloidosis in the USA. *Nephrology Dialysis Transplantation*. 2022;37(12):2569-2580. doi:10.1093/ndt/gfac196

2.	Sawinski D, Lim MA, Cohen JB, et al. Patient and Kidney Allograft Survival in Recipients With End-Stage Renal Disease From Amyloidosis. *Transplantation*. 2018;102(2)

3.	Tang W, McDonald SP, Hawley CM, et al. End-stage renal failure due to amyloidosis: outcomes in 490 ANZDATA registry cases. *Nephrol Dial Transplant*. Feb 2013;28(2):455-61. doi:10.1093/ndt/gfs492

4.	Yamauchi J, Raghavan D, Jweehan D, et al. Kidney Transplant Outcomes in Amyloidosis: US National Database Study. *Transplantation*. Mar 1 2025;109(3):536-548. doi:10.1097/TP.0000000000005191

5.	Angel-Korman A, Stern L, Sarosiek S, et al. Long-term outcome of kidney transplantation in AL amyloidosis. *Kidney International*. 2019;95(2):405-411. doi:10.1016/j.kint.2018.09.021

6.	Bergesio F, Ciciani AM, Manganaro M, et al. Renal involvement in systemic amyloidosis *: An Italian collaborative study on survival and renal outcome. *Nephrology Dialysis Transplantation*. 2008;23(3):941-951. doi:10.1093/ndt/gfm684

7.	Bollee G, Guery B, Joly D, et al. Presentation and Outcome of Patients with Systemic Amyloidosis Undergoing Dialysis. *Clinical Journal of the American Society of Nephrology*. 2008;3(2)

8.	Cohen OC, Law S, Lachmann HJ, et al. The impact and importance of achieving a complete haematological response prior to renal transplantation in AL amyloidosis. *Blood Cancer Journal*. 2020/05/22 2020;10(5):60. doi:10.1038/s41408-020-0325-2

9.	Decourt A, Gondouin B, Delaroziere JC, et al. Trends in Survival and Renal Recovery in Patients with Multiple Myeloma or Light-Chain Amyloidosis on Chronic Dialysis. *Clinical Journal of the American Society of Nephrology*. 2016;11(3)

10.	Gertz MA, Leung N, Lacy MQ, et al. Clinical outcome of immunoglobulin light chain amyloidosis affecting the kidney. *Nephrology Dialysis Transplantation*. 2009;24(10):3132-3137. doi:10.1093/ndt/gfp201

11.	Gertz MA, Kyle RA, O'Fallon WM. Dialysis Support of Patients With Primary Systemic Amyloidosis: A Study of 211 Patients. *Archives of Internal Medicine*. 1992;152(11):2245-2250. doi:10.1001/archinte.1992.00400230061010

12.	Havasi A, Heybeli C, Leung N, et al. Outcomes of renal transplantation in patients with AL amyloidosis: an international collaboration through The International Kidney and Monoclonal Gammopathy Research Group. *Blood Cancer Journal*. 2022/08/18 2022;12(8):119. doi:10.1038/s41408-022-00714-5

13.	Herrmann SMS, Gertz MA, Stegall MD, et al. Long-term outcomes of patients with light chain amyloidosis (AL) after renal transplantation with or without stem cell transplantation. *Nephrology Dialysis Transplantation*. 2011;26(6):2032-2036. doi:10.1093/ndt/gfr067

14.	Heybeli C, Bentall A, Wen J, et al. A study from The Mayo Clinic evaluated long-term outcomes of kidney transplantation in patients with immunoglobulin light chain amyloidosis. *Kidney International*. 2021/03/01/ 2021;99(3):707-715. doi:<https://doi.org/10.1016/j.kint.2020.06.036>

15.	Law S, Cohen O, Lachmann HJ, et al. Renal transplant outcomes in amyloidosis. *Nephrology Dialysis Transplantation*. 2021;36(2):355-365. doi:10.1093/ndt/gfaa293

16.	Leung N, Kumar SK, Glavey SV, et al. The impact of dialysis on the survival of patients with immunoglobulin light chain (AL) amyloidosis undergoing autologous stem cell transplantation. *Nephrology Dialysis Transplantation*. 2016;31(8):1284-1289. doi:10.1093/ndt/gfv328

17.	Pinney JH, Lachmann HJ, Sattianayagam PT, et al. Renal Transplantation in Systemic Amyloidosis—Importance of Amyloid Fibril Type and Precursor Protein Abundance. *American Journal of Transplantation*. 2013/02/01/ 2013;13(2):433-441. doi:<https://doi.org/10.1111/j.1600-6143.2012.04326.x>

18.	Sattianayagam PT, Gibbs SDJ, Pinney JH, et al. Solid Organ Transplantation in AL Amyloidosis. *American Journal of Transplantation*. 2010/09/01/ 2010;10(9):2124-2131. doi:<https://doi.org/10.1111/j.1600-6143.2010.03227.x>

19.	Abedi AS, Nakhjavani JM, Etemadi J. Long-Term Outcome of Renal Transplantation in Patients With Familial Mediterranean Fever Amyloidosis: A Single-Center Experience. *Transplantation Proceedings*. 2013/12/01/ 2013;45(10):3502-3504. doi:<https://doi.org/10.1016/j.transproceed.2013.09.021>

20.	Altindal M, Turkmen E, Yildirim T, et al. Kidney transplantation for end-stage renal disease secondary to familial Mediterranean fever. *Clinical Transplantation*. 2016/07/01 2016;30(7):787-790. doi:<https://doi.org/10.1111/ctr.12749>

21.	Bitik B, Hatipoğlu B, Sayın B, et al. Long-term results of kidney transplantation in patients with familial mediterranean fever. *Clinical Transplantation*. 2023/02/01 2023;37(2):e14888. doi:<https://doi.org/10.1111/ctr.14888>

22.	Emiroglu R, Başaran Ö, Pehlivan S, et al. Effect of Amyloidosis on Long-Term Survival in Kidney Transplantation. *Transplantation Proceedings*. 2005/09/01/ 2005;37(7):2967-2968. doi:<https://doi.org/10.1016/j.transproceed.2005.07.010>

23.	Gursu M, Yelken B, Caliskan Y, et al. Outcome of Patients with Amyloidosis after Renal Transplantation: A Single-Center Experience. *The International Journal of Artificial Organs*. 2012/06/01 2012;35(6):444-449. doi:10.5301/ijao.5000091

24.	Haq A, Hussain S, Meskat B, Mohan P, Conlon P, Hickey DP. Complications of Renal Transplantation in Patients With Amyloidosis. *Transplantation Proceedings*. 2007/01/01/ 2007;39(1):120-124. doi:<https://doi.org/10.1016/j.transproceed.2006.10.021>

25.	Keven K, Sengul S, Kutlay S, et al. Long-term outcome of renal transplantation in patients with familial Mediterranean fever amyloidosis: A single-center experience. *Transplantation Proceedings*. 2004/11/01/ 2004;36(9):2632-2634. doi:<https://doi.org/10.1016/j.transproceed.2004.09.065>

26.	Koc Y, Basturk T, Unsal A, et al. Effects of AA Amyloidosis on Survival in Peritoneal Dialysis. *Kidney and Blood Pressure Research*. 2012;36(1):182-190. doi:10.1159/000343407

27.	Kofman T, Grimbert P, Canoui-Poitrine F, et al. Renal transplantation in patients with AA amyloidosis nephropathy: results from a French multicenter study. *Am J Transplant*. Nov 2011;11(11):2423-31. doi:10.1111/j.1600-6143.2011.03620.x

28.	Kuroda T, Tanabe N, Sato H, et al. Outcome of patients with reactive amyloidosis associated with rheumatoid arthritis in dialysis treatment. *Rheumatology International*. 2006/10/01 2006;26(12):1147-1153. doi:10.1007/s00296-006-0204-6

29.	Özdemir BH, Özdemir FN, Sezer S, Şar A, Haberal M. Among Therapy Modalities of End-Stage Renal Disease, Renal Transplantation Improves Survival in Patients With Amyloidosis. *Transplantation Proceedings*. 2006/03/01/ 2006;38(2):432-434. doi:<https://doi.org/10.1016/j.transproceed.2005.12.051>

30.	Özdemir BH, Özdemir FN, Demirhan B, Turan M, Haberal M. Renal transplantation in amyloidosis: effects of HLA matching and donor type on recurrence of primary disease. *Transplant International*. 2004/06/01 2004;17(5):241-246. doi:<https://doi.org/10.1111/j.1432-2277.2004.tb00437.x>

31.	Sahutoglu T, Atay K, Caliskan Y, Kara E, Yazici H, Turkmen A. Comparative Analysis of Outcomes of Kidney Transplantation in Patients With AA Amyloidosis and Chronic Glomerulonephritis. *Transplantation Proceedings*. 2016/07/01/ 2016;48(6):2011-2016. doi:<https://doi.org/10.1016/j.transproceed.2016.04.015>

32.	Sarihan I, Caliskan Y, Mirioglu S, et al. Amyloid A Amyloidosis After Renal Transplantation: An Important Cause of Mortality. *Transplantation*. Aug 2020;104(8):1703-1711. doi:10.1097/TP.0000000000003043

33.	Sarıtaş H, Sendogan DO, Kumru G, et al. Long-term Results of Kidney Transplantation in Patients With Familial Mediterranean Fever and Amyloidosis. *Transplantation Proceedings*. 2019/09/01/ 2019;51(7):2289-2291. doi:<https://doi.org/10.1016/j.transproceed.2019.04.074>

34.	Schwarz C, Georgin-Lavialle S, Lombardi Y, et al. Kidney Transplantation in Patients With AA Amyloidosis: Outcomes in a French Multicenter Cohort. *American Journal of Kidney Diseases*. 2024;83(3):329-339. doi:10.1053/j.ajkd.2023.07.020

35.	Sherif AM, Refaie AF, Sobh MA-K, Mohamed NA-H, Sheashaa HA, Ghoneim MA. Long-term outcome of live donor kidney transplantation for renal amyloidosis. *American Journal of Kidney Diseases*. 2003/08/01/ 2003;42(2):370-375. doi:<https://doi.org/10.1016/S0272-6386(03)00676-0>

36.	Tatar E, Karatas M, Aykas A, Okut G, Bozkaya G, Uslu A. Infections After Renal Transplant in Recipients With Familial Mediterranean Fever: A Life-Threatening Issue. *Exp Clin Transplant*. Feb 2017;15(Suppl 1):240-243. doi:10.6002/ect.mesot2016.P113

37.	Ylinen K, Gronhagen-Riska C, Honkanen E, Ekstrand A, Metsarinne K, Kuhlback B. Outcome of patients with secondary amyloidosis in dialysis treatment. *Nephrology Dialysis Transplantation*. 1992;7:908-912.

38.	Gillmore JD, Stangou AJ, Lachmann HJ, et al. Organ transplantation in hereditary apolipoprotein AI amyloidosis. *Am J Transplant*. Oct 2006;6(10):2342-7. doi:10.1111/j.1600-6143.2006.01507.x

39.	Gillmore JD, Lachmann Hj Fau - Rowczenio D, Rowczenio D Fau - Gilbertson JA, et al. Diagnosis, pathogenesis, treatment, and prognosis of hereditary fibrinogen A alpha-chain amyloidosis. 2009;(1533-3450 (Electronic))

40.	Meyer L, Ulrich M, Ducloux D, et al. Organ Transplantation in Hereditary Fibrinogen A alpha-Chain Amyloidosis: A Case Series of French Patients. *Am J Kidney Dis*. Sep 2020;76(3):384-391. doi:10.1053/j.ajkd.2020.02.445
